# Urban environment decreases pollinator availability, fertility, and prolongs anthesis in the field bindweed (*Convolvulus arvensis* Linnaeus, 1753)

**DOI:** 10.1080/15592324.2024.2325225

**Published:** 2024-03-06

**Authors:** Pavol Prokop

**Affiliations:** a Department of Environmental Ecology and Landscape Management, Faculty of Natural Sciences, Comenius University, Bratislava, Slovakia; b Institute of Zoology, Slovak Academy of Sciences, Bratislava, Slovakia

**Keywords:** Behavioural plasticity, flowering duration, urban pollinators

## Abstract

Urbanization alters the natural environment, with broad negative impacts on living organisms. Urbanization can also disrupt plant-pollinator networks by reducing the abundance and diversity of invertebrates. Firstly, I investigated whether the field bindweed (*Convolvulus arvensis*) is an obligatory entomophilous plant because previous reports were ambiguous. Secondly, I investigated how the obligatory entomophilous plant, field bindweed, responds to urbanization by comparing the flowering duration (anthesis) and the reproductive success of field bindweeds in urban and rural populations. Unlike cross-pollinated flowers and controls, flowers experimentally prevented from pollination and self-pollinated flowers did not produce seeds, suggesting that the field bindweed is self-incompatible and obligatory entomophilous. The abundance of urban pollinators was 5–6 times lower than the abundance of rural pollinators, and flies (Diptera), beetles (Coleoptera) and moths (Lepidoptera) were significantly more negatively influenced by the urban environment than hymenopterans (Hymenoptera). Urban plants showed significantly longer anthesis duration and lower reproductive success than rural plants. Illuminance and low pollinator abundance were negatively associated with the duration of the anthesis, but relative humidity did not affect the anthesis. Prolonged duration of the anthesis may be an adaptation to pollinator scarcity because more prolonged flowering increases the likelihood of pollination. Future research should unravel whether the longer anthesis of urban flowers is determined by behavioral plasticity or by the evolutionary selection of plants with a genetically determined longer anthesis.

## Introduction

Urbanization, rapid expansion, and development of urban areas are responsible for environmental, evolutionary, and behavioral change.^1,2^ By creating barriers that isolate species populations of living organisms, the urban environment contributes to habitat loss and fragmentation,^3,4^ negatively influencing gene flow.^5^ Specific environmental conditions in cities (e.g., higher temperatures, elevated air, noise, and light pollution) further contribute to a decrease in the diversity and abundance of native plants^6^ and animals.^7,8^ Adaptations to an urban environment have led to selection of traits that confer a fitness advantage.^9,10^ Unfortunately, the impact of cities on living organisms is expected to be more intense in the near future because the rate of global expansion of urbanization has unprecedently expanded by 9,687 km^2^ per year.^11^

A major issue with urbanization is its negative impact on pollinator richness, disrupting native plant – pollinator networks,^12–14^ although not to the same extent as intensified agricultural landscapes.^15^ The diversity of pollinators can be higher in urban than in agricultural areas^16^ and these differences could be mainly due to changes in species phenology rather than abundance.^17^ However, the impact of urbanization also depends on the taxonomic groups for which entomophilous plants are adapted for; flies (Diptera) and butterflies (Lepidoptera) are more vulnerable to negative anthropogenic impacts than hymenoptera.^14,18,19^ Although pollinators in cities could be positively supported by the high diversity and abundance of flowering plants and landscape heterogeneity (the Urban Pollinator Enrichment hypothesis), habitat fragmentation, invasive species, pollen limitation, and artificial light have profoundly negative effects on pollinators and pollination (the Urban Pollinator Deficit Hypothesis^20–23^ If we consider native plants rather than exotic ones, I suggest that urban pollinator abundance and diversity should be lower in cities due to the above arguments.

Plants respond to environmental stimuli through a range of responses and adaptive strategies that interested Darwin^24^ more than 100 years ago. These behaviors seem to be very sensitive to urbanization; for example, summer blooming plants occurring in high human population densities are associated with extended flowering duration.^25^ Flowering duration is a result of complicated interactions involving abiotic (temperature [urban heat effect, climate change,^25^ relative humidity and illumination,^26,27^ and biotic factors (pollinator availability.^26,28–36^ Flowering is costly,^29,37^ thus a reduction of flowering duration prevents additional physiological costs associated with flowering duration. Altered flowering and pollinator phenology driven by urbanization can have a negative effect on the fitness of plant communities and change food webs in ecosystems.^38,39^ Global analyzes of how urbanization impacts flowering phenology and duration^25,40^ did not examine possible associations between pollinator availability and flowering duration.

In this study, I examined the effects of the urban environment on the fitness of the field bindweed (*Convolvulus arvensis* Linnaeus, 1753). *C. arvensis* responds to pollinator availability by reducing flowering time^41^ which makes this species a sensitive indicator on pollinator occurrence. My first goal was to reexamine the putative self-incompatibility of the field bindweed, which could be questioned, given that experimental self-pollination and cross-pollination resulted in zero seed set^41^ which was interpreted as possible self-pollination.^42^ This issue is crucial because only the dependence on pollinators makes this species a suitable indicator of pollinator availability and, ultimately, environmental change. My second goal was to test the urban pollinator deficit hypothesis, suggesting that low pollinator abundance and richness negatively influence individual fitness of entomophilous plants. I predict that plants in an urban environment have a longer flowering time than plants in a rural environment, because prolonged flowering increases the likelihood of being pollinated, particularly in environments with low pollinator abundance. Furthermore, I predict that urban plants have lower fertility than plants in a rural environment due to inadequate pollinator services. I hypothesize that the predicted prolonged flowering time in urban plants remains a disadvantage. Human-induced rapid environmental change (HIREC^43^ might occur too rapidly, hindering the spread of sufficient adaptations.

## Methods

### Study species

The field bindweed (Convolvulaceae) is a common climbing perennial plant native to the Europe and Asia, which grows in a wide range of conditions. It is probably insect-dependent and self-incompatible.^41,44–47^ Most plants in Slovakia bloom between June and September (pers. obs.). Radially symmetrical white (sometimes pink) flowers open early in the morning and close in the afternoon, but absence of pollination can prolong flowering during the night and the next day for up to 42 hours.^41^ Flowers are visited by honeybees, bumblebees, halictid bees, butterflies, and moths.^44–47^ The ovary has four basally attached ovules.^48^ The number of seeds per capsule varies between one and four.^49^

### Study site

The experiments were carried out in uncut urban and rural habitats in and near Trnava, Slovakia (N 48^○^37′, E 17^○^58′), from 23 June to 27 July 2023. For the anthesis duration experiment, I randomly selected three uncut plots in the city center (N 48^○^382′, E 17^○^591′, N 48^○^380′, E17^○^592′ and N 48^○^374′, E 17^○^588′) and three uncut plots in rural habitats (N 48^○^396′, E 17^○^583′, N 48^○^395′, E 17^○^575′ and N 48^○^397′, E 17^○^572′). For this research, I defined the urban environment as the environment densely surrounded by buildings in the city. In contrast, the rural environment was defined as the environment near the city directly connected to open fields and agricultural land. Only flowers close to the ground, rather than flowers that curl on tall plants, were preferred to standardize the experimental conditions. Typical surrounding plants were *Achillea vulgaris, Ballota nigra, Cirsium arvense, Cichorium intybus, Crepis setosa, Securigera varia, Echium vulgare, Melilotus officinalis, Sonchus asper, Tripleurospermum inodorum*, and others.

### Experimental procedure

#### The self-incompatibility experiment

The self-incompatibility experiment was carried out on an additional uncut experimental plot in the rural area near Trnava (N 48^○^395′, E 17^○^574′). On the day of the experiment, still closed flowers were randomly assigned to one of four treatments: self-pollinated (*N* = 18), cross-pollinated (*N* = 19), non-pollinated (*N* = 21) and control (*N* = 19). All flowers were individually marked with a ribbon before opening between 06:00 and 07:30 hours on 14 July 2023. Except for the control group, each flower was bagged using fine mesh. This mesh served to prevent any interaction between the flowers and pollinators. The tissue was carefully affixed to the stem of each flower using a plastic clip. The use of a fine brush did not yield seed production in this species.^41^ To perform self-pollination, I gently grabbed a stamen with soft tweezers, bent it, and rubbed an anther on the stigma. To perform hand-cross pollination, I used anthers from flower heads collected at least 10 m apart from focal plants and rubbed the stigmas of focal plants in the same way as in self-pollination. The tweezers were carefully cleaned to remove pollen after each pollination. The experiment was carried out in the morning when anthers release pollen (P. Prokop, pers. obs.). I checked the presence of mesh bags on experimental flowers daily and the tissue was removed 48 hours later. The seeds were finally collected and counted 13–14 days after the experiment.

#### The anthesis duration experiment

##### Pilot study

**Figure 1.**
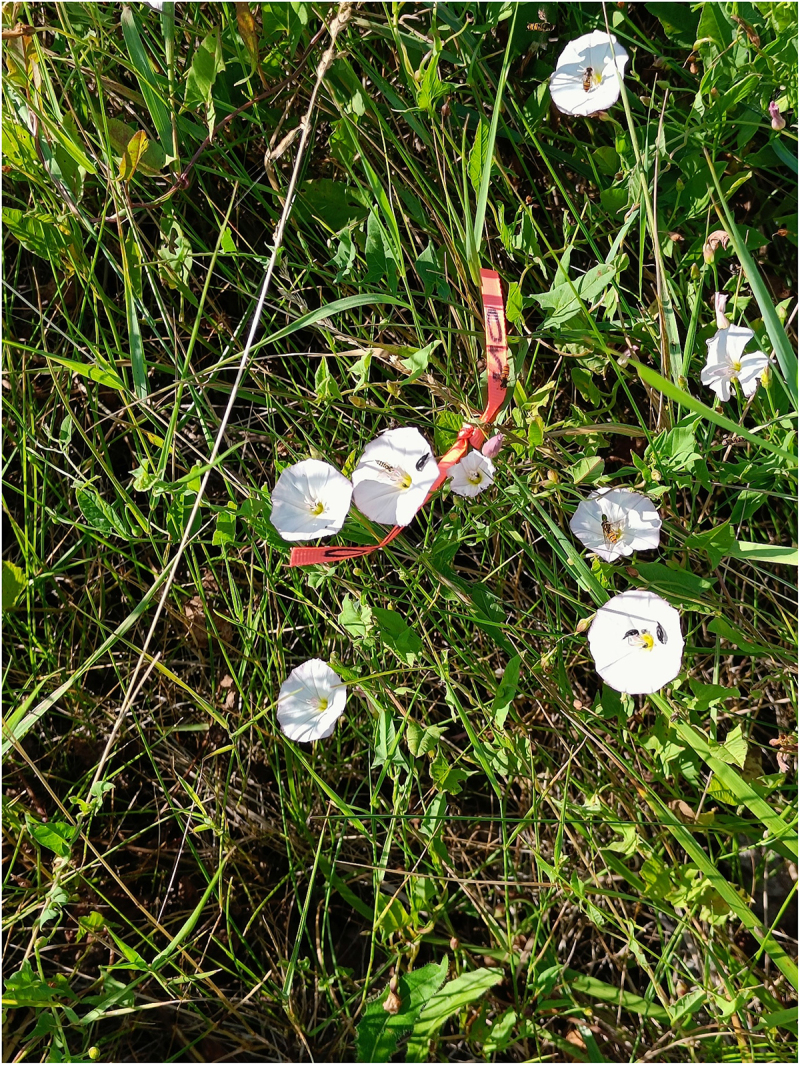
Individually marked plant in rural area with a high abundance of syrphid flies and beetles (*Podonta nigrita*).

The pilot study was carried out on 11 and 12 August 2021. I selected five regularly cut plots with *C. arvensis* in the urban area and five plots in the rural area. Semi-opened flowers at least 1 m apart from each other were individually marked with a ribbon at 07:30 and pollinators were recorded each 30 min throughout the day until the final capitulum was closed (Figure 1).

##### Main study

The main study was conducted in the summer of 2023. Compared to the pilot study, it was elaborated to record of the duration of the anthesis and additional measurements described below. On the day of the experiment, 30 individual semi-opened flowers per plot were marked with a ribbon. Each flower belonged to a different plant, and the distance between the marked flowers was 1 m. Individual marking took place approximately between 06:30 and 06:50 AM. At 07:00 AM, I measured natural illumination (i.e., natural light falling on the internal surface of the corolla) with a Kimo LX 100lux meter. The lux meter was placed directly above the flower. Relative humidity (RH) near each flower and overall temperature in the experimental plot were measured with a Voltcraft DL-220THP datalogger. Temperature was measured only once in the same manner as RH near a randomly selected flower per plot. The number of neighboring flowers was measured by counting all neighboring flowers in a radius of 12.5 cm. This size was self-determined on the basis on my preliminary observations, suggesting that this radius was found to provide a representative sample size of surrounding flowers. The occurrence of pollinators was recorded by observing pollinators between 07:30 and 10:00 when their numbers peak (P. Prokop, pers. obs.). I repeatedly observed 2–5 flowers simultaneously for 4 min and then moved on to other flowers. The identification of pollinators was conducted primarily at the level of insect orders. In particular, pollinators that visited the flowers most frequently were photographed and identified at the species level. Pollination was defined, following Patiño et al.,^50^ as an event when insects fed on flowers continuously for at least three seconds. Each flower was observed approximately 20 min. At 10:00 AM, the measurements of light (on each flower), relative humidity (near each flower), and overall temperature (in each plot) were repeated. The flowers were then checked every 30 min until their corollas were closed (hereafter anthesis duration). I have chosen sunny and windless days with less than 50% cloud cover and air temperatures exceeding 25°C to minimize environmental effects on pollinator behavior. The reproductive success of each flower was determined by calculating the total number of seeds produced. Seed collection and counting were performed 12 days after the experiment was conducted.

### Statistical analyses

Kruskal-Wallis ANOVA was applied in the self-incompatibility experiment because the data contained many zero values and were not normally distributed. A simple comparison of the abundance of pollinators between the urban and rural area was performed with a generalized linear model (GLM) with Poisson distribution of the data. Path analysis was used to examine associations between anthesis duration and environmental variables (illumination, relative humidity) or ecological variables (urban versus rural environment, number of flowers in 12.5 cm radius, total number of pollinators) influenced the duration of the anthesis. Three direct associations were defined based on *p* < .05 from the preparatory regression analysis. (1) Anthesis duration (dependent variable) associated with the urban/rural environment, illumination, and pollinator abundance. (2) Pollinator abundance (dependent variable) associated with the urban/rural environment and illumination. (3) RH (dependent variable) associated with the urban/rural environment and illumination. The total number of flowers in 12.5 cm radius never had a significant association with dependent variables and was not included in the path model. The root mean square error of approximation (RMSEA), the standardised root mean square residual (SRMR) and the Comparative Fit Index (CFI) and were used to assess model fit for path analyses. The reproductive success of each flower (number of seeds) was defined as a dependent variable in the generalised linear model (GLM) with Poisson distribution of the data. The interaction terms were not significant and were therefore not included to analyses. Simple comparisons of the number of neighboring flowers, humidity, and light intensity between urban and rural environments were compared with the Mann-Whitney U test. The abundance of all major pollinator groups between urban and rural environments was compared with the chi-square test (for *N* > 120) or with Fisher’s exact test (for *N* < 120). I preferred to use light and relative humidity data measured at 10:00 (data obtained at 07:00 were omitted), because they showed stronger correlations with dependent variables. Most statistical tests were performed with Statistica ver. 12.0. Path analysis was performed with Jamovi ver. 2.4.11.^51^

## Results

### The self-incompatibility experiment

**Figure 2.**
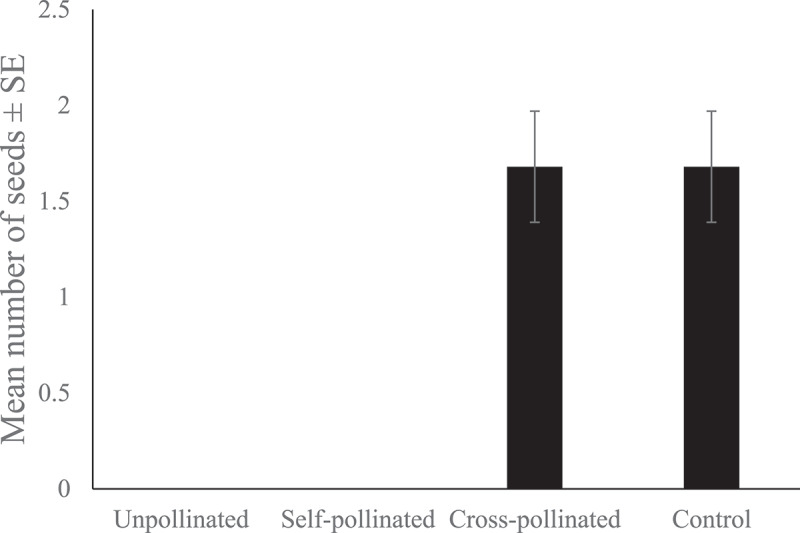
Differences in seed production across four treatments.

There were significant differences in the total number of seeds between treatments (Kruskal-Wallis χ2 = 26.7, df = 3, *p* < .001). Cross-pollinated flowers produced an identical number of seeds as control flowers. Unpollinated and self-pollinated flowers did not produce seeds (Figure 2).

### The anthesis duration experiment

#### Pilot study

Of the 70 pollinators observed in the rural area, Diptera (mainly Syrphidae) were most abundant (*N* = 32, 45.7%), followed by Lepidoptera (mainly Noctuidae) (*N* = 19, 27.1%), Hymenoptera (*N* = 16, 22.9%) and Coleoptera (*N* = 3, 4.3%). On the contrary, the urban area (*N* = 17 pollinators) was composed mainly of Hymenoptera (mainly Halictidae) (*N* = 16, 94.1%), followed by the observation of a syrphid fly (5.9%). The differences in the abundance of pollinators belonging to the main orders of insect between urban and rural environments were significant (2 × 4 Fisher exact test, *p* < .001). The abundance of pollinators in the urban area (mean = 0.28, SE = 0.12, *N* = 61) was significantly lower than in the rural area (mean = 1.67, SE = 0.12, *N* = 60) (GLM, estimate = 0.72, Wald χ2 = 28.04, *p* < .001).

#### Main study

Three flowers in rural habitats were predated by *Leptophyes albovittata* (Orthoptera: Tettigoniidae) and five flowers did not open completely. These flowers were removed from further statistical analyzes. None of these flowers produced seeds.

### Anthesis duration

**Figure 3.**
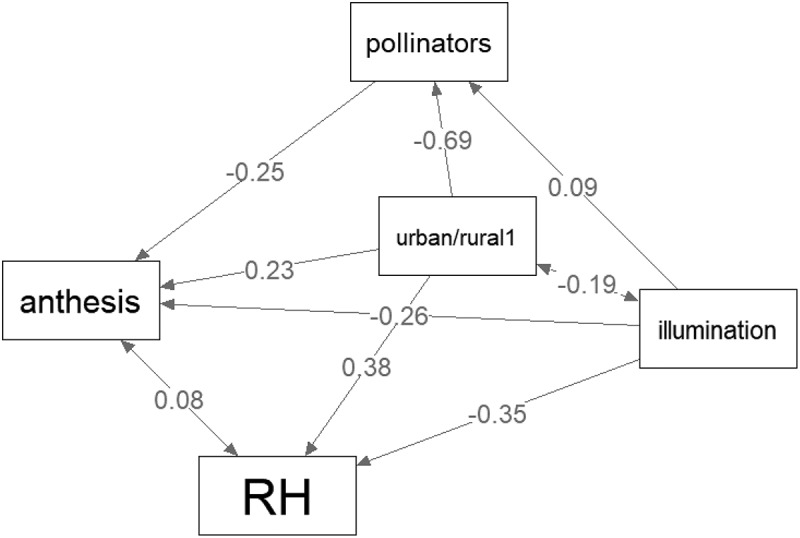
Graphic display of the estimated path analysis model. The numbers are beta values. For statistical significance, see the text.

Model fit criteria indicated that the path analysis model had a good fit (CFI >0.99, RMSEA < 0.05 and SRMR = 0.01). The anthesis of urban flowers was significantly longer (mean = 520 min, SE = 8.34, N = 90) than the anthesis of rural flowers (mean = 439 min, SE = 8.74, N = 82) (p = .009, Figure 3). Urban areas showed a significantly higher number of neighboring flowers, higher humidity, and lower light intensity than rural areas (Table 1). A higher abundance of pollinator was significantly associated with a shorter anthesis (p = .005). The more intense illumination at 10:00 AM was significantly associated with shortened anthesis (p < .001) (Figure 3).

**Table 1.** Descriptive statistics for environmental differences between the urban and rural environment compared with the M-W U-test.

	Number (N) of neighboring flowers	RH (%)	Illumination (kilolux)
Urban (*N* = 90)	3.54 (0.33)	49.53 (0.99)	54.49 (3.74)
Rural (*N* = 82)	2.17 (0.35)	40.14 (1.04)	68.81 (3.92)
U	2567.5	1787	2921
P	<.001	<.001	<.05

### Pollinator abundance

Of the 171 pollinators observed in both urban and rural environments, the majority (*N* = 73, 42.7%) were flies (mainly Syrphidae, e.g., *Episyrphus balteatus*, less Bombyliidae, e.g., *Bombylisoma unicolor*), followed by hymenopterans (*N* = 49, 28.7%, mainly Halictidae) and beetles (*N* = 46, 26.9%, e.g., *Podonta nigrita*, *Oedemera femorata*). Moths (*Acontia luctuosa* and *Emmelia trabealia*) and bugs (Heteroptera) were rarely observed (*N* = 2 and 1, respectively). Comparison of the main insect groups (flies, beetles, hymenopterans) revealed that their occurrence between urban and rural environments differs significantly (2 × 3 Chi-square test, χ2 = 27.53, *p* < .001). Most flies (65/73, 89%) and beetles (40/46, 87%) were recorded in the rural environment, while hymenopterans occurred in both environments equally (25/49, 51% in the rural environment). Moths and bugs were observed exclusively in rural environment but were omitted from statistical analyzes due to low sample sizes. Interestingly, while the honeybee *Apis mellifera* was observed on focal plant in rural areas only once (1/25, 4% of all rural hymenopterans), it was significantly more willing to visit bindweeds in urban areas (17/24, 70.8% of all hymenopterans) (Fisher exact test, *p* < .001). Path analysis revealed that the total number of pollinators observed in flowering bindweeds was significantly lower in urban habitats (mean = 0.57, SE = 0.15, *N* = 90) than in rural habitats (Mean = 3.23, SE = 0.16, *N* = 82) (*p* < .001). More illuminated flowers tended to be visited by more pollinators than less illuminated flowers, although this association was not significant (*p* = .09, Figure 3).

### Relative humidity

RH was significantly higher in urban areas than in rural areas (*p* < .001) and was negatively correlated with illuminance (*p* < .001) (Figure 3).

### Fertility

30.9% (22 out of 71) and 42.2% (38 of 90) seed abortions were observed in the rural and urban area, respectively (χ^2^ = 0.70, *p* = 0.41). GLM showed that plants were more fertile in the rural environment (*M* = 1.79, SE = 0.17, *N* = 71) than in the urban environment (*M* = 1.27, SE = 0.15, *N* = 90) and that a greater number of neighboring flowers positively influenced fertility (Table 2).

**Table 2.** GLM on fertility.

	Estimate	SE	Wald χ^2^	± 95% CI	P
Intercept	−0.37	0.59	0.39	−1.52, 0.79	.53
Neighbouring flowers	0.04	0.02	4.98	0.005, 0.08	.03
Illumination	0.00	0.002	0.006	−0.004, 0.004	.94
RH	0.003	0.008	0.15	−0.012, 0.02	.70
Pollinator abundance	0.23	0.30	0.60	−0.36, 0.82	.44
Anthesis	0.001	0.001	1.10	−0.0007, 0.003	.29
Urban/Rural	0.21	0.10	4.39	0.01, 0.40	.04

## Discussion

The first objective of this study was to reexamine the putative self-incompatibility of field bindweed using a more appropriate methodology than previously used. The second objective was to examine the negative effect of urban environment on plant behavior and individual fitness. The field bindweed received fewer pollinators and showed lower fertility in urban areas compared to rural areas, accordance to the Urban Pollinator Deficit Hypothesis. Plants found in the urban environment showed prolonged anthesis and lower seed sets compared to plants in rural areas.

The first experiment that investigated pollinator availability showed that field bindweed is self-incompatible and strongly pollinator dependent. The experimental plants (but not the untreated control) in the previous research failed to produce seeds regardless of the type of treatment (self-pollinated, cross-pollinated or non-pollinated). These results could be interpreted in various ways.^42^ For instance, the field bindweed could be perceived as self-incompatible because pollinator-prevented flowers did not produce seeds.^41^ However, infertility between treated flowers does not allow us to reject the hypothesis that this species is self-incompatible. Therefore, the current research, where I did not use brush, which appeared to be very invasive for field bindweed stigma, clearly confirmed previous suspicions by Prokop and Neupauerová^41^ regarding self-incompatibility. Notably, during my afternoon control (15:30) of flowers in the 1^st^ day of the experiment, 84% of control flowers were closed, while most of non-pollinated flowers (81%) remained still open. This difference was strongly significant (Fisher exact test, *p* < .001) and further corroborates the idea that the field bindweed responds to pollination visitation by shortening the duration of the anthesis. Because previous research has been carried out in an urban environment^41^ and the current experiment was performed in a rural environment, these results appear to be applicable to both environments.

The Urban Pollinator Deficit Hypothesis^20^ suggests that low pollinator abundance and richness compromise the individual fitness of entomophilous plants. Two predictions derived from this hypothesis. First, urban field bindweed showed a longer anthesis than flowers in a rural environment. Prolonged anthesis is suggested to be an adaptation to pollinator scarcity.^28,32–35,41^ Both pilot and main studies conducted in different years showed that the urban environment is characterized by significantly lower pollinator abundance than the rural environment, which could contribute to a prolonged flowering time.

Although the pollinator examination methodology was not uniform, the results were very consistent; the pilot study and the main study showed that the abundance of pollinators was 5.9× and 5.7× times higher in the rural environment than in the urban environment, respectively. Although I did not exactly examine the species richness, the occurrence of pollinators belonging to insect orders consistently showed that flies, beetles, and moths were significantly more common in the rural environment, whereas hymenopterans were distributed equally across environments. These results corroborate recent findings showing that flies and butterflies are more vulnerable to negative anthropogenic impacts than Hymenoptera.^14,18,19^

Except for the effect of urbanization, the duration of the anthesis was negatively influenced by the intensity of the illumination, but not by the relative humidity. Light is tightly associated with temperature and humidity,^27^ and an interaction of these environmental factors influences the circadian clock.^52^ These factors need to be addressed under well-controlled laboratory conditions. However, flowers of many plants exhibit a growth or movement response in relation to the direction of sunlight (heliotropism) and altered exposure to sunlight improves pollinator visitation.^53^ Perhaps, the more exposed field bindweed flowers were more intensively pollinated and therefore reduced the overall flowering time.

According to the second prediction, urban flowers showed a reduced seed set compared to rural flowers, which provides further support for the Urban Pollinator Deficit Hypotheses. The decrease in seed production can be attributed to a decrease in pollinator abundance, but I found no statistical support for this possibility. This decline in pollinator abundance may be a consequence of the reduced availability of suitable sites for insect pollinators to nest and forage in urban environment, as indicated by various studies.^18,54,55^ A higher number of neighboring flowers was associated with higher fertility, suggesting that the flowers attracted more pollinators.^14,56^

Although pollinator abundance was higher in rural than urban environments, the reproductive success of urban plants was not drastically compromised. The low abundance of urban pollinators that still pollinated a significant number of flowers was possibly outweighed by high abundance of generalist insect pollinators (e.g., halictid bees) and managed bee species (i.e., honey bees)^14,23^ and/or by changes in foraging preferences of these pollinators. For instance, halictid bees were very common in urban areas and these bees show strong preferences for field bindweed flowers.^47^ Perhaps halictid flies could provide pollination services similar to urban field bindweeds as spiral horned bees (e.g., *Systropha curvicornis*) specialized in field bindweed pollination, but that occur exclusively in rural areas. However, I observed bees on competing plants in rural habitats very frequently, but field bindweed flowers were largely ignored. On the contrary, urban honeybees frequently visited field bindweeds, despite similar competing plant species occurring also in urban areas.

Although both predictions of the of urban pollinator deficiency hypothesis were supported, crucial influences of pollinator visitation on the duration of anthesis were significant, but the association between pollinator abundance and plant reproductive success was not statistically confirmed. One probable explanation is that the pollinator observation time was not sufficient to explain most of the variability of the results obtained. Further research can use camera traps to obtain a complete picture of all pollinators visiting focal flowers, although it should be noted that other researchers use a similar time to observe pollinators.^42,57^ Not all pollinators provide adequate pollinator service to a given plant species, thus visitation rates cannot precisely influence the reproductive success. Another explanation is that, although the field bindweed shows apparent behavioral plasticity of anthesis duration in response to pollinator visitation,^41^ it is still possible that the urban environment favors genes that support prolonged anthesis duration, while no similar selection occurs in the rural environment, where pollinators are abundant. This latter scenario does not predict a necessary correlation between individual’s anthesis duration or reproductive success and pollinator visitation. These two possibilities need to be disentangled by examining genetic correlations in anthesis duration in urban and rural environment.

In conclusion, the urban environment influences plant behavior in terms of prolonged anthesis duration and reduced reproductive success, which could be caused by plant behavioral plasticity or by evolutionary processes favoring plants with longer flowering durations. Because pollinators alter anthesis duration in many plant species, pollinator availability (besides other biotic and abiotic variables) should be considered in future studies modeling the impact of urbanization on plant fitness.
